# What makes Americans fall behind in their finances? Evidence from the national well-being survey

**DOI:** 10.3389/fpsyg.2022.1087418

**Published:** 2023-01-09

**Authors:** Laura Raisa Miloș, Marius Cristian Miloș, Flavia Mirela Barna, Claudiu Boțoc

**Affiliations:** Department of Finance, Faculty of Economics and Business Administration, West University of Timisoara, Timisoara, Romania

**Keywords:** financial well-being, financial hardship, ordered logits, United States, financial knowledge

## Abstract

The question of how often Americans fall behind on their finances is analyzed using the National Financial Well-Being survey of the United States Consumer Financial Protection Bureau. An ordered logit model is proposed to study the effect of individual and household characteristics on the likelihood of falling behind in one’s finances. The analysis shows that traditional variables such as income, age, education, and health are statistically significant predictors of falling behind in one’s finances. In addition, the study shows that the volatility of income, saving habits, and individuals’ financial knowledge significantly contribute to the explanation of Americans’ economic behavior.

## 1. Introduction

In 2016, the United States Consumer Financial Protection Bureau (CFPB) conducted the National Financial Well-Being survey to collect United States. household information and situational measures to construct financial well-being (FWB) scores, ranging between 0 and 100. According to a CFPB report (CFPB, 2017), 90 percent of individuals with FWB scores ranging between 11 and 40 (low FWB scores) expressed hardship in making ends meet. On the other end of the spectrum, only 10 percent of individuals with FWB scores greater than 60 reported having difficulty making ends meet (CFPB, 2017). In addition, the descriptive analysis of the report also indicates that as the individuals’ FWB score increases, their financial security increases.

An increasing body of studies deals with self-reported happiness, also termed life satisfaction or subjective well-being (SWB).^1^ The growing interest in subjective well-being finds its explanation in advocates calling to use the SWB as a substitute or complement to traditional income-based welfare measures. The reasoning behind this approach is that subjective well-being is positive not only for individuals, leading to labor market performance and willingness to invest in the future (Graham, 2016) but also for society as a whole, leading to economic growth and social welfare (DiMaria et al., 2020), to labor market performance and willingness.

A limited body of research has investigated financial hardship among older Americans as a means to improve policy decision-making (Bierman, 2014; Wilkinson, 2016; Marshall et al., 2021). In this paper, we use the question “How often would you say I am behind with my finances?” from the FWB survey to identify and estimate the effect of the variables that lead American adults to believe they are behind with their finances. We follow previous literature using variables such as income, age, number of children in the household, marital/partnership status, and health status as the main determinants in forming the FWB scores. To our knowledge, this is the first study that uses the National Financial Well-Being Survey and goes beyond the descriptive analysis of the CFPB.

## 2. Theoretical background

The assessment of subjective well-being (SWB) in general and financial well-being in particular and their implications in welfare measurement is crucial for policymakers and academia in different research areas, specifically economics, personal financial planning, and psychology. In the policy arena, some policymakers have started advocating using the SWB as a substitute or complement to traditional indicators or measures of welfare. For example, in the Commission on the Measurement of Economic and Performance and Social Progress reported to the French President, Nicolas Sarkozy, Stiglitz et al. (2009) stipulate that GDP, a market activity indicator, is neither a measure of income nor a measure of well-being. Therefore, the authors recognize the need to go beyond measures of market activity to measures of well-being and the need to devise statistical tools and metrics to measure well-being and societal progress appropriately. In the same line of thought, Helliwell (2006) concludes, using international well-being data, that well-being measures could be used in individual welfare measurement. However, the author warns against the fact that several well-being studies establish linkages or correlations but not necessarily causation between policy initiatives or variables and well-being measures. Bache and Reardon (2017) place subjective well-being both as an objective of public policy and as a way of measuring its impact.

In terms of public policy impact on subjective well-being, Di Tella et al. (2001) used data from 12 European countries and the United States to conclude that lower unemployment and inflation rates are linked with high levels of reported well-being. On the margin, the unemployment rate negatively impacts reported well-being more than inflation. The study shows that individuals are willing to trade off a one percentage point increase in the unemployment rate for a 1.7 percentage point increase in the inflation rate, suggesting the misery index^2^ underestimates the unhappiness caused by unemployment. This finding corroborates Frey and Stutzer (2000), who found that the unemployment rate has a depressing effect on subjective well-being, while institutional factors positively influence subjective well-being.

Researchers have approached subjective well-being under two different assumptions in academia: the cardinality assumption and the ordinality assumption. In psychology, most studies assume SWB scores to be cardinal; that is, the difference between a score of 5 and a score of 4 is the same as the difference between a score of 9 and a score of 8 (Ferrer-i-Carbonell and Frijters, 2004). This assumption leads psychologists to use simple estimation tools, such as ordinary least squares (OLS), to model the SWB. In contrast, most economists (see Jenkins, 2020, for example) assume the SWB scores to be ordinal. Under this assumption, the difference between an SWB score of 5 and a score of 4 is not necessarily equal to the difference between a score of 9 and 8. Consequently, economists use the latent variable approach to explain the variation in the SWB scores. Another stream of research bridges the gap between the two methodological differences by setting up and estimating a latent variable model with individual fixed effects, taking advantage of the availability of panel data surveys of SWB.

Despite the methodological differences between these approaches, the main determinants of SWB have been classified by Dolan et al. (2008) into seven categories: (1) income or some variants of it, (2) personal characteristics (age, gender, ethnicity, and personality), (3) socially developed characteristics (education, health, type of work, and unemployment), (4) time allocation (hours worked, commuting, caring for others, community involvement and volunteering, exercise, and religious activities), (5) attitudes toward self/others/life (attitudes toward our circumstances, trust, political persuasion, and religion), (6) relationships (marriage and intimate relationships, having children, seeing family and friends), and (7) economic, social, and political environment (income inequality, unemployment rates, inflation rate, welfare system and public insurance, degree of democracy, climate and the natural environment, safety and deprivation of the area, and urbanization).

Although individuals, regardless of their socioeconomic status, culture and religion, and localization, share some common approaches to overall life satisfaction, subjective well-being comprehends different satisfactions: financial happiness, health satisfaction, family satisfaction, and work satisfaction. Most previous studies focus on the issue of subjective well-being as measured by answering the general question, “How happy are you at present with your life as a whole?” using a scale from 0 to 10 or 1 to 10. One of the main criticisms of the subjective well-being approach is its inability to provide an appropriate measure of life satisfaction and happiness, given its dependence on the conditions and context under which individuals are asked to answer the question.

This paper departs from the previous literature by analyzing the determinants of financial well-being (FWB) as measured by the National Financial Well-Being survey carried on by the Consumer Financial Protection Bureau (CFPB) in 2016. We argue that although the FWB survey scores contain some elements of subjectivity, the questions asked to the survey respondents are more specific and objective. Our rationale for using the question “How often would you say I am behind with my finances?” as the dependent variable instead of the overall FWB score is that this variable is not affected by the environment, mindset, or mood. Instead, it constitutes a precise financial statement about the respondent’s financial health. We strongly believe that financial well-being offers a mixture of objective indicators and subjective perceptions and reactions to oneself financial situation.

## 3. Data and methodology

In 2016, The Consumer Financial Protection Bureau conducted the first nationwide financial well-being of adult Americans to construct a financial well-being scale as well as collect other variables, including individual characteristics, household and family characteristics, income and employment characteristics, savings and safety nets, financial experiences, and financial behaviors, skills, and attitudes. The survey data consist of 6,394 observations, of which 5,395 observations are from the general population sample, and 999 observations are from the oversample of those aged 62 or older. The survey uses weights to ensure the sample represents the United States. population across geographic and demographic dimensions.

The data contains the CFPB financial well-being scale constructed from 10 questions related to different aspects of the respondent’s financial life (see CFPB, 2017 for a detailed description of the data). In addition, the data consist of (1) individual characteristics, including age, education, physical health, ethnicity, and gender; (2) household and family characteristics, such as housing status, marital status, and financially supporting children; (3) income and employment characteristics, including information on employment status, household income, federal poverty status, and income volatility; (4) savings and safety nets, including liquid savings, ability to absorb an unexpected expense, non-retirement investments, health insurance, and the availability of friends/family safety net for emergency needs; (5) financial experiences with credit rejection, debt collection, checking or savings account; use of non-bank short-term credit, use of non-bank transaction product, housing cost burden, negative financial services experience, experience with any adverse financial shocks, student loan, financial socialization, and responsibility for own finances; and (6) financial behaviors, skills, and attitudes, such as confidence in ability to achieve a financial goal, a habit of saving, effective day-to-day money management behaviors, planning horizon of 5 and more years, propensity to plan for finances, financial knowledge, and financial skills. Table 1 provides descriptive statistics of the variables used in the analysis.

**Table 1:** Frequency distribution of the categorical variables.

Variable		Frequency	Percent
I am behind with my finances	Never	2,427	39.56
	Rarely	2,018	32.89
	Sometimes	1,042	16.98
	Often	396	6.56
	Always	252	4.11
Age categories	18–24	390	6.36
	25–34	1,066	17.38
	35–44	793	12.93
	45–54	1,030	16.79
	55–61	681	11.10
	62–69	986	16.07
	70–74	487	7.94
	> 74	702	11.44
Gender	Female	2,909	47.42
	Male	3,226	52.58
Education	Less than high school	398	6.49
	High school degree/GED	1,548	25.23
	Some college/Associate degree	1,859	30.30
	Bachelor’s degree	1,274	20.77
	Graduate/Professional degree	1,056	17.21
Household size	1	1,176	19.17
	2	2,612	42.58
	3	950	15.48
	4	779	12.70
	>4	618	10.07
Partnership status	Married	3,681	60.00
	Widowed	347	5.66
	Divorced/Separated	657	10.71
	Never married	1,093	17.82
	Living with partner	357	5.82
Ethnicity	White	4,347	70.86
	Black, non-Hispanic	636	10.37
	Other, non-Hispanic	326	5.31
	Hispanic	826	13.46
Income	< $20,000	669	10.90
	$20,000–$29,999	484	7.89
	$30,000–$39,999	585	9.54
	$40,000–$49,999	444	7.24
	$50,000–$59,999	493	8.04
	$60,000–$74,999	626	10.20
	$75,000–$99,999	920	15.00
	$100,000–$149,999	1,078	17.57
	> $150,000	836	13.63
Income volatility	Roughly the same each month	4,474	72.93
	Roughly the same most months	1,263	20.59
	Often varies quite a bit from one month to the next	398	6.49
Health status	Poor	147	2.40
	Fair	751	12.24
	Good	2,069	33.72
	Very good	2,501	40.77
	Excellent	667	10.87
Housing status	I own	4,041	65.87
	I rent	1,576	25.69
	I do not currently own or rent	518	8.44
Employment	Self-employed	402	6.55
	Work full-time	2,461	40.11
	Work part-time	423	6.89
	Homemaker	352	5.74
	Full-time student	217	3.54
	Permanently sick, disabled, or unable to work	261	4.25
	Unemployed or temporarily laid off	232	3.78
	Retired	1,787	29.13
Saving habits	Strongly disagree	288	4.69
	Disagree	591	9.63
	Disagree slightly	628	10.24
	Agree slightly	1,270	20.70
	Agree	1,671	27.24
	Strongly agree	1,687	27.50
Financial knowledge	1 to very low	117	1.91
	2	144	2.35
	3	507	8.26
	4	1,430	23.31
	5	2,580	42.05
	6	1,045	17.03
	7 to very high	312	5.09

Own elaboration.

We assume that the statement” I am behind with my finances” posed to the survey respondents measures their financial struggles. Thus, we follow previous literature in using variables such as income, age, gender, education, household size, ethnicity, marital, health, housing, employment status, employment volatility, saving habits, and financial knowledge as the main determinants in the formation of the FWB scores, in general, and in answering that statement, in particular. Table 1 provides descriptive statistics of the variables used in the analysis. As can be seen, all variables included in the study are categorical, and a parameter estimate is obtained for each category.

The empirical approach assumes that respondents scale or score different questions in an ordered way. We assume there is a correct but unobserved or latent score or index, say $y_{i}^{*}$, that measures the financial well-being of the individuals. Furthermore, we assume that this latent score is a linear function of the variables described above and collected in the vector $x_{i}$, that is,


(1) $$y_{i}^{*}=x_{i}^{\prime }\beta +\varepsilon_{i}$$

where β is the vector of parameters to be estimated, and $\varepsilon_{i}$ represents the error terms. As the latent variable or index crosses some unknown threshold, we observe the scores are moving up according to the following expression


(2) $$\{\begin{array}{c} \begin{array}{c} y_{i}=1\;if\;y_{i}^{*}\leq \lambda_{1}\;\\ y_{i}=2\;if\;\lambda_{1}<y_{i}^{*}\leq \lambda_{2}\\ y_{i}=3\;if\;\lambda_{2}<y_{i}^{*}\leq \lambda_{3} \end{array}\\ \vdots \\ y_{i}=J\;if\;\lambda_{J-1}<y_{i}^{*} \end{array}$$

The model is completed by specifying the distribution of the error terms. If we assume the error terms are identical and independently distributed according to the logistic distribution, that is $F\;\left(\lambda_{j}-x_{i}^{\prime }\beta \right)=\frac{\exp\left(\lambda_{j}-x_{i}^{\prime }\beta \right)}{1+\exp\left(\lambda_{j}-x_{i}^{\prime }\beta \right)}=\wedge \;\left(\lambda_{j}-x_{i}^{\prime }\beta \right)$; we obtain the ordered logit given by


$$\{\begin{array}{c} \mathrm{Pr}\left[y_{i}=j\right]=\frac{\exp\left(\lambda_{j}-x_{i}^{\prime }\beta \right)}{1+\exp\left(\lambda_{j}-x_{i}^{\prime }\beta \right)}-\frac{\exp\left(\lambda_{j-1}-x_{i}^{\prime }\beta \right)}{1+\exp\left(\lambda_{j-1}-x_{i}^{\prime }\beta \right)}\;\left(3\right)\\ =\wedge \;\left(\lambda_{j}-x_{i}^{\prime }\beta \right)-\wedge \;\left(\lambda_{j-1}-x_{i}^{\prime }\beta \right) \end{array}$$

The effect of the covariates $x_{i}$ on the probability of observing a given score is obtained using the partial effect given by


(4) $$\frac{\partial \;\mathrm{Pr}\left[y_{i}=\lambda_{j}\right]}{\partial x_{i}}=\left[\wedge^{\prime }\left(\lambda_{j}-x_{i}^{\prime }\beta \right)-\wedge^{\prime }\left(\lambda_{j-1}-x_{i}^{\prime }\beta \right)\right]\beta$$

where $\wedge^{\prime }$ = ∧(1 − ∧) is the derivative of ∧.

Regarding the estimation issues, we acknowledge there might be a feedback or simultaneity effect between financial well-being and some variables, such as health status, financial knowledge, and education. However, the issue is beyond this analysis’s scope due to the data’s cross-sectionality. We include several control variables to palliate this endogeneity problem.

## 4. Results and discussion

Table 2 summarizes the results of the estimation of equation (3). Overall, the model is statistically significant, evidenced by a high $\chi^{2}$ value and a very low *p*-value compared to the model without explanatory variables (null model). In addition, most of the variables included in the model are statistically significant at different significance levels. We did not provide the cutoff points to avoid having cumbersome result tables. In addition, the cutoff points do not offer some meaningful interpretation, hence we did not include them in our discussion.

**Table 2:** Ordered logit estimation results.

Variable		Estimate	Std. error	Value of *p*
Age categories	18–24	Base		
	25–34	0.0784	0.1481	0.597
	35–44	−0.0866	0.1618	0.592
	45–54	−0.1458	0.1607	0.364
	55–61	−0.3737**	0.1719	0.030
	62–69	−0.5379***	0.1802	0.003
	70–74	−0.8015***	0.2073	0.000
	>74	−0.7398***	0.2138	0.001
Gender	Male	Base		
	Female	−0.0740	0.0600	0.217
Education	Less than high school	Base		
	High school degree/GED	0.0022	0.1278	0.986
	Some college/Associate degree	0.2769**	0.1312	0.035
	Bachelor’s degree	0.2499*	0.1410	0.076
	Graduate/Professional degree	0.3589**	0.1485	0.016
Household size	1	Base		
	2	0.0112	0.0946	0.906
	3	0.1666	0.1106	0.132
	4	0.2327**	0.1186	0.050
	> 4	0.2688**	0.1245	0.031
Partnership status	Married	Base		
	Widowed	0.0429	0.1480	0.772
	Divorced/Separated	0.1687	0.1035	0.103
	Never married	0.0116	0.1070	0.913
	Living with partner	0.0198	0.1340	0.882
Ethnicity	White	Base		
	Black, non-Hispanic	0.5194***	0.0972	0.000
	Other, non-Hispanic	0.3297**	0.1291	0.011
	Hispanic	0.0668	0.0873	0.444
Income	< $20,000	Base		
	$20,000–$29,999	−0.3686***	0.1391	0.008
	$30,000–$39,999	−0.4934***	0.1303	0.000
	$40,000–$49,999	−0.5460***	0.1569	0.000
	$50,000–$59,999	−0.6542***	0.1432	0.000
	$60,000–$74,999	−0.7622***	0.1295	0.000
	$75,000–$99,999	−0.7785***	0.1285	0.000
	$100,000–$149,999	−0.8847***	0.1306	0.000
	>$150,000	−1.1409***	0.1479	0.000
Income volatility	Roughly the same each month	Base		
	Roughly the same most months, with some unusual high or low	0.3609***	0.0670	0.000
	Often varies quite a bit from 1 month to the next	0.7860***	0.1215	0.000
Health status	Poor	Base		
	Fair	−0.3157	0.2238	0.158
	Good	−0.6414***	0.2176	0.003
	Very good	−0.8471***	0.2193	0.000
	Excellent	−1.2815***	0.2376	0.000
Housing status	I own	Base		
	I rent	0.3860***	0.0785	0.000
	I do not currently own or rent	0.0246	0.1297	0.850
Employment	Self-employed	Base		
	Work full-time	0.0672	0.1196	0.575
	Work part-time	0.0322	0.1506	0.831
	Homemaker	−0.1283	0.1627	0.430
	Full-time student	0.3410*	0.2008	0.089
	Permanently sick, disabled, or unable to work	0.0509	0.1939	0.793
	Unemployed or temporarily laid off	0.4895**	0.2040	0.016
	Retired	−0.3500**	0.1408	0.013
Saving habits	Strongly disagree	Base		
	Disagree	−0.4104**	0.2006	0.041
	Disagree slightly	−0.5847***	0.1955	0.003
	Agree slightly	−0.9976***	0.1913	0.000
	Agree	−1.5094***	0.1948	0.000
	Strongly agree	−2.1286***	0.2011	0.000
Financial knowledge	1 to very low	Base		
	2	−0.4245	0.3388	0.210
	3	−0.0632	0.2740	0.817
	4	−0.4242	0.2624	0.106
	5	−0.7850***	0.2625	0.003
	6	−0.9867***	0.2737	0.000
	7 to very high	−1.0340***	0.3168	0.000

(*)10% significance level (**)5% significance level (***)1% significance level; se: estimate’s standard error. Own elaboration.

For the age category, the results indicate that as we move up in the age category (higher age), the likelihood of an individual struggling financially decreases. Recall that for this question, the lower the score is (1 for never and 5 for always), the lower the chance of falling behind with one’s finances. Unlike age, the gender variable is not statistically significant, implying there is no difference between males and females in explaining the likelihood of lagging in one’s finances. At the same time, the variable education yields surprising results as the analysis shows that as the level of education increases, the likelihood of falling behind in finance increases. This might be due to the level of student debt after graduation, and further research is worthwhile to explore this linkage. On the other hand, as one would expect, financial struggles increase as the household size increases. The results also indicate that the likelihood of struggling financially for black non-Hispanic and other non-Hispanic races is more significant than for whites. However, there is no statistical difference between whites and Hispanics regarding this issue.

This analysis also confirms previous studies finding that income is statistically significant in explaining an individual’s financial well-being (Zyphur et al., 2015). In addition, our results show that as we move up in the income categories, the probability of financial struggle diminishes, increasing as income volatility increases. Thus, individuals with roughly the same monthly income are less likely to fall behind in their finances. This is also the case for individuals with excellent health, for whom the results indicate that they are less likely to have problems with their finances than less healthy individuals.

Furthermore, individuals living in rental housing are more likely to experience financial hardship than those owning their houses, *ceteris paribus*. While for the partnership or marital status, the results show that these variables are not statistically significant.

Our results prove that these variables are statistically significant in explaining the variation in the likelihood of financial struggles in saving habits and financial knowledge. This likelihood decreases as individuals are strongly prone to save more. The same effect is observed for the financial knowledge variable. The results show that their financial struggles decrease as the individual’s financial knowledge increases.

However, other than the statistical significance and the direction of the effect, Table 2 results do not provide any information regarding the magnitude of the impacts of different variables on the likelihood of individuals falling or never falling behind in their finances. The marginal effects presented in Table 3 provide a quantification of the impact of each category for each variable. Without loss of generality, we emphasize interpreting the results for the likelihood of often and always falling behind in one’s finances. Hence, for the variable age categories, the results show a non-statistically significant difference between age categories 18–24, 25–34, 35–44, and 45–54. However, as we increase in the age categories, the likelihood of never falling behind in one’s finances increases. For example, individuals in the age categories 55–61, 62–69, 70–74, and greater than 74 are, respectively, 6.53, 9.52, 14.43, and 13.28% more likely to never fall behind in their finances compared to the age category 18–24. The same age categories are 3.38, 4.66, 6.28, and 6.05%, less likely to often and always fall behind with their finances.

**Table 3:** Ordered logit marginal effect (me) estimates.

Variables		I am behind with my finances										Never		Rarely		Sometimes		Often		Always		ME estimate	se	ME estimate	se	ME estimate	se	ME estimate	se	ME estimate	se
Age categories	18–24	Base group									
	25–34	−0.0130	0.0276	−0.0006	0.0010	0.0056	0.0107	0.0041	0.0076	0.0039	0.0073
	35–44	0.0147	0.0273	0.0001	0.0007	−0.0064	0.0119	−0.0044	0.0083	−0.0041	0.0077
	45–54	0.0489	0.0272	−0.0001	0.0008	−0.0108	0.0118	−0.0073	0.0082	−0.0067	0.0076
	55–61	0.0653**	0.0296	−0.0034*	0.0019	−0.0281**	0.0129	−0.0180**	0.0089	−0.0158**	0.0077
	62–69	0.0952***	0.0315	−0.0080***	0.0029	−0.0407***	0.0136	−0.0251***	0.0089	−0.0215***	0.0078
	70–74	0.1443***	0.0372	−0.0191***	0.0064	−0.0607***	0.0157	−0.0354***	0.0096	−0.0291***	0.0080
	> 74	0.1328***	0.0383	−0.0161***	0.0062	−0.0561***	0.0163	−0.0331***	0.0099	−0.0274***	0.0083
Gender	Male	Base group									
	Female	0.0127	0.0103	−0.0007	0.0006	−0.0053	0.0043	−0.0035	0.0028	−0.0032	0.0026
Education	Less than high school										
	High school degree/GED	−0.0004	0.0222	0.0001	0.0026	0.0002	0.0090	0.0001	0.0056	0.0001	0.0050
	Some college/Associate	−0.0472***	0.0226	0.0033	0.0025	0.0192*	0.0091	0.0128**	0.0058	0.0119**	0.0054
	Bachelor’s degree	−0.0427*	0.0242	0.0032	0.0025	0.0174*	0.0097	0.0115*	0.0063	0.0106*	0.0058
	Graduate degree	−0.0607**	0.0252	0.0034	0.0025	0.0247**	0.0101	0.0168**	0.0068	0.0159**	0.0065
Household size	1	Base group									
	2	−0.0020	0.0166	0.0002	0.0016	0.0008	0.0069	0.0005	0.0043	0.0004	0.0037
	3	−0.0288	0.0192	0.0020	0.0015	0.0120	0.0080	0.0078	0.0052	0.0007	0.0046
	4	−0.0400*	0.0204	0.0022	0.0015	0.0167*	0.0085	0.0110*	0.0056	0.0100*	0.0051
	> 4	−0.0461**	0.0213	0.0023	0.0014	0.0193**	0.0089	0.0128**	0.0060	0.0117**	0.0055
Partnership status	Married	Base group									
	Widowed	−0.0074	0.0254	0.0004	0.0013	0.0031	0.0106	0.0020	0.0070	0.0018	0.0064
	Divorced/Separated	−0.0287*	0.0174	0.0010*	0.0005	0.0120	0.0073	0.0081	0.0051	0.0075	0.0048
	Never married	−0.0020	0.0184	0.0001	0.0012	0.0008	0.0077	0.0005	0.0050	0.0005	0.0045
	Living with partner	−0.0034	0.0230	0.0002	0.0014	0.0014	0.0096	0.0009	0.0063	0.0008	0.0057
Ethnicity	White	Base group									
	Black, non-Hispanic	−0.0869***	0.0156	−0.0005	0.0019	0.0366**	0.0068	0.0260***	0.0053	0.0248***	0.0054
	Other, non-Hispanic	−0.0563***	0.0214	0.0019*	0.0010	0.0238***	0.0091	0.0161	0.0067	0.0146**	0.0064
	Hispanic	−0.0117	0.0252	0.0010	0.0012	0.0049	0.0064	0.0031	0.0041	0.0027	0.0036
Income	<$20,000	Base group									
	$20,000–$29,999	0.0573***	0.0218	0.0105**	0.0044	−0.0257***	0.0099	−0.0212***	0.0080	−0.0208***	0.0079
	$30,000–$39,999	0.0781***	0.0206	0.0117***	0.0044	−0.0352***	0.0094	−0.0279***	0.0075	−0.0267***	0.0073
	$40,000–$49,999	0.0871***	0.0255	0.0118***	0.0044	−0.0393***	0.0118	−0.0306***	0.0089	−0.0290***	0.0082
	$50,000–$59,999	0.1059***	0.0234	0.0114**	0.0045	−0.0478***	0.0108	−0.0360***	0.0082	−0.0335***	0.0075
	$60,000–$74,999	0.1251***	0.0209	0.0101**	0.0046	−0.0564***	0.0098	−0.0412***	0.0075	−0.0376***	0.0070
	$75,000–$99,999	0.1281***	0.0206	0.0099**	0.0045	−0.0577***	0.0098	−0.0420***	0.0076	−0.0382***	0.0070
	$100,000–$149,999	0.1474***	0.0212	0.0076*	0.0045	−0.0663***	0.0102	−0.0468***	0.0077	−0.0418***	0.0070
	>$150,000	0.1981***	0.0251	−0.0013	0.0052	−0.0869***	0.0119	−0.0574***	0.0082	−0.0494***	0.0072
Income volatility	Roughly the same each month	Base group									
	Roughly the same most months, with some unusual high or low	−0.0622***	0.0117	0.0023***	0.0009	0.0269***	0.0052	0.0176***	0.0034	0.0154***	0.0032
	Often varies quite a bit from 1 month to the next	−0.1288***	0.0183	−0.0069	0.0043	0.0552***	0.0081	0.0410***	0.0072	0.0394***	0.0007
Health status	Poor	Base group									
	Fair	0.0474	0.0323	0.0105	0.0096	−0.0202	0.0135	−0.0183	0.0133	−0.0195	0.0150
	Good	0.1011***	0.0313	0.0139	0.0096	−0.0440***	0.0132	−0.0356***	0.0130	−0.0355**	0.0147
	Very good	0.1371***	0.0318	0.0119	0.0096	−0.0598***	0.0137	−0.0455***	0.0132	−0.0437***	0.0148
	Excellent	0.2168***	0.0364	−0.0020	0.0105	−0.0938***	0.0158	−0.0637***	0.0137	−0.0573***	0.0150
Housing status	I own	Base group									
	I rent	−0.0663***	0.0436	0.0017*	0.0010	0.0288***	0.0062	0.0190***	0.0040	0.0169***	0.0036
	I do not currently own or rent	−0.0044	0.0230	0.0004	0.0022	0.0019	0.0099	0.0011	0.0060	0.0009	0.0049
Employment	Self-employed	Base group									
	Work full-time	−0.0117	0.0209	0.0006	0.0012	0.0050	0.0089	0.0033	0.0058	0.0029	0.0050
	Work part-time	−0.0056	0.0262	0.0003	0.0014	0.0024	0.0112	0.0016	0.0073	0.0013	0.0063
	Homemaker	0.0227	0.0286	−0.0019	0.0026	−0.0096	0.0122	−0.0060	0.0076	−0.0051	0.0064
	Full-time student	−0.0575	0.0333	−0.0006	0.0026	0.0246	0.0143	0.0173	0.0104	0.0161	0.0100
	Permanently sick, disabled, or unable to work	−0.0089	0.0337	0.0004	0.0016	0.0038	0.0144	0.0025	0.0094	0.0021	0.0083
	Unemployed or temporarily laid off	−0.0810**	0.0329	−0.0035	0.0045	0.0346**	0.0141	0.0254**	0.0110	0.0245**	0.0111
	Retired	0.0628**	0.0252	−0.0080**	0.0034	−0.0265**	0.0107	−0.0156**	0.0064	−0.0127**	0.0054
Saving habits	Strongly disagree	Base group									
	Disagree	0.0500**	0.0228	0.0356*	0.0192	−0.0243**	0.0105	−0.0296**	0.0147	−0.0316*	0.0169
	Disagree slightly	0.0745***	0.0223	0.0470**	0.0189	−0.0376***	0.0104	−0.0413***	0.0145	−0.0423***	0.0165
	Agree slightly	0.1408***	0.0218	0.0626***	0.0189	−0.0736***	0.0107	−0.0666***	0.0145	−0.0631***	0.0161
	Agree	0.2363***	0.0230	0.0580***	0.0190	−0.1218***	0.0121	−0.0918***	0.0148	−0.0807***	0.0161
	Strongly agree	0.3634***	0.0252	0.0197	0.0170	−0.1761***	0.0134	−0.1134***	0.0150	−0.0937***	0.0161
Financial knowledge	1 to very low	Base group									
	2	0.0674	0.0534	0.0099	0.0103	−0.0304	0.0239	−0.0238	0.0191	−0.0231	0.0192
	3	0.0095	0.0407	0.0023	0.0106	−0.0042	0.0180	−0.0037	0.0162	0.0040	0.0172
	4	0.0674*	0.0391	0.0100	0.0102	−0.0304	0.0174	−0.0238	0.0155	0.0231	0.0164
	5	0.1308***	0.0394	0.0072	0.0103	−0.0588***	0.0177	−0.0415***	0.0156	−0.0378**	0.0164
	6	0.1681***	0.0420	0.0015	0.0105	−0.0750***	0.0190	−0.0502***	0.0159	−0.0443***	0.0166
	7 to very high	0.1769***	0.0516	−0.0003	0.0122	−0.0788***	0.0230	−0.0521***	0.0172	−0.0457***	0.0171

(*)10% significance level (**)5% significance level (***)1% significance level; se: estimate’s standard error. Own elaboration

The education variable yields interesting results. In theory, education provides the background for making informed and rational decisions regarding personal finance matters and financial risk management (Maman and Rosenhek, 2019). However, our results show overall that as the education level increases, the probability of experiencing financial hardship increases. For instance, individuals with some college or associate degree are 2.47% more likely to often or always endure financial hardship and 4.72% less likely to never fall behind in their finances than individuals with less than a high school diploma. On the other hand, individuals with a bachelor’s degree are 4.27% less likely to never fall behind in their finances and 2.21% more likely to express they are often or always behind in their finances than the base group (individuals with less than high school). On the other hand, individuals with a graduate or professional degree are 6.07% less likely to ever fall behind in their finances. However, they are 4.72% less likely to have a high school degree or General Education Development (GED). A potential explanation for these findings could be the high cost of student loan repayments, as suggested in the case of the United Kingdom by Mccloud and Bann (2019) or the desire to attain and maintain a social lifestyle.

In terms of household type and size, the estimation results indicate that marital or partnership status does not affect the difference in the likelihood of falling behind finances between the different partnership statuses, contrasting previous evidence found in the literature (Gorman, 2000). Hence, there is no statistically significant difference between married, widowed, divorced or separated, never married, and living with a partner in the probability of often or always falling behind the finances. However, a household with four persons or more are 4.55% more likely to express that they often or always fall behind in their finances than a household with only one person. On the other hand, households with four persons or more are 8.61% less likely to ever fall behind in their finances.

Furthermore, our results indicate that the ethnicity/race variable is essential in explaining the differences in the likelihood of falling behind in finances. Hence, non-Hispanic black individuals are 8.69% less likely to never fall behind in their finances than white individuals. The results also show that non-Hispanic blacks are 5.08% more likely to often or always express that they are behind with their finances. Other non-Hispanic races are 3.07% more likely than whites to often or always fall behind with their finances and 5.63% less likely never to feel financial hardship than white individuals. The results partially confirm the conclusions of Bhutta et al. (2020), which show substantial disparities between United States White and Black families’ wealth, posing threats to their financial security. At the same time, there is no statistical difference between white and Hispanic individuals in the likelihood of falling behind in their finances. Lee and Hanna (2012) show, for instance, that although black individuals and Hispanic individuals have similar characteristics in terms of low homeownership rates, income or net worth, there are some differences in the predicted financial delinquency rates (such as debt payment problems). Hispanic households have lower predicted delinquency rates in comparison with black households and even white households. According to Lee and Hanna (2012), Hispanics might behave differently than other groups due to several internal and external reasons related to their upbringing, cultural beliefs and attitudes. However, other studies have pointed out that Hispanic Americans have been one of the most negatively affected groups after the housing crisis and the Great Recession (Porto, 2016). More personal finance research on the topic is necessary.

Unlike previous studies, we model income using the level and volatility of income for the income variables. Overall, both the income level and the volatility variables are statistically significant in explaining the probability of falling behind with one’s finances. For the income levels, as we move up in the income categories, the probability of never falling behind increases, while the probability of often or always falling behind on finances decreases. Hence, individuals in the $20,000–$29,999 income bracket are 5.73% more likely to never fall behind with their finances than individuals with income less than $20,000. Likewise, there is a 74% chance that individuals in the $100,000–$149,999 income bracket never fall behind their finances compared to individuals with less than $20,000 income.

Individuals with more than $150,000 are more likely to never fall behind with their finances than individuals with less than $20,000 income. On the other hand, individuals with less than $20,000 annual income are more likely to often or always fall behind on their finances than individuals with a yearly revenue of $100,000 or higher. Holding the income level the same, individuals with income that often varies are 8.04% more likely to often or always fall behind on their finances than individuals with roughly the same monthly income.

Similar to the income variables, the health status variables are also statistically highly significant in explaining the likelihood of individuals often falling behind on their finances. Hence, compared to individuals who self-assess as having poor health, individuals who self-assess as having excellent health are 21.68% more likely to never fall behind on their finances. Conversely, the figure drops to 13.71 and 10.11% for individuals who self-assess as having very good and good health, respectively. On the other end of the spectrum, there are 12.10, 8.92, and 7.11% fewer chances to often or always fall behind with their finances for individuals with excellent, very good, and good health, respectively, compared to individuals with poor health.

In addition, individuals who rent are 3.59% more likely to experience financial hardship by often or always falling behind with their finances than individuals who own their houses. The latter is also 6.63% more likely to never fall behind with their finances than those renting. Besides, unemployed or temporarily laid-off individuals are 4.99% more likely to often or always fall behind their finances than self-employed individuals, who are 8.10% more likely never financially to fall behind when compared to unemployed or temporarily laid-off individuals. We observe an opposing pattern for retired as they are 6.28% more likely never to endure financial hardship and 2.83% less likely to often or always fall behind in their finances than self-employed individuals. There is no statistically significant difference between other employment categories (full-time, part-time, homemaker, and full-time students) and self-employed individuals in their likelihood of enduring financial hardship.

In terms of statistical and economic significance, one of the critical findings of this study is the effect of saving habit variables on the likelihood of enduring financial hardship. The results show that individuals’ likelihood of never falling behind with their finances increases as the scale perception about saving increases. Hence, individuals who strongly agree with saving habits are 36.34% more likely to never fall behind with their finances than individuals who strongly disagree with having them. The figure decreases to 23.63, 14.08, 7.45, and 5% for individuals who agree, slightly agree, slightly disagree, and disagree, respectively. When it comes to often or always falling behind with finances, individuals who agree or strongly agree with saving habits are 37.96% less likely to endure financial hardship than individuals who strongly disapprove of saving habits. The saving habits could fall into the category of actions with delayed gratification taken for longer-term well-being. The ability to delay gratification enables individuals to give greater weight to the investment component of lifestyle decisions (Gschwandtner et al., 2022). Our findings are consistent with the literature that sees immediate or current gratification as a key inhibitor of financial effectiveness (Vyvyan et al., 2014). A balance between spending and saving is healthy for financial well-being in the longer run (Van Praag et al., 2003; Sehravat et al., 2021).

Likewise, individuals with high and very high financial knowledge are 34.50% more likely to never fall behind with their finances. In comparison, individuals with average to good knowledge are 19.82% more likely never to endure financial hardship than individuals with very low financial knowledge. The results also indicate that having a high to very high financial knowledge decreases the probability of often or always falling behind with finances by 19.23%, compared to individuals with very low financial knowledge.

In sum, this study yields important findings with a significant impact on the economic policy implications. First, the results show that improving Americans’ saving habits reduces the likelihood of falling behind with one’s finances. According to a Pew Research Center survey, 63% of Americans admit they are not saving enough (Taylor et al., 2016). The United States household saving rate in 2016 (the National Financial Well-Being survey year) averaged 6.7% of disposable household income, lower than 18.7% in Switzerland, 16.0% in Sweden, 13.7% in France, and 9.8% in Germany but comparable to 6.7% in the United Kingdom (Organisation for Economic Co-operation and Development, 2021).

Second, the results indicate that improving United States. individuals’ financial literacy will reduce the probability of often or always falling behind in finances. In this aspect, there is still considerable room for improvement in the United States compared to other countries in the OECD. According to the S&P Global Financial Literacy Survey realized, in 2014, 57% of adult Americans were considered financially literate, compared to 71% in Norway, Denmark, and Sweden, or 68% in Canada and Israel. In addition, the adoption of financial literacy in the public education system could equip individuals with financial knowledge and skills and improve their attitudes and behaviors to make adequate financial decisions (Contreras and Bendix, 2021). Lastly, health status is another area where appropriate policy decisions can reduce the likelihood of financial hardship among Americans. According to our results, individuals with very good or excellent health are 35.39% more likely to never fall behind with their finances.

## 5. Conclusion

This paper analyzes how often adult Americans fall behind on their finances using the National Financial Well-Being survey of the United States Consumer Financial Protection Bureau. An order logit model is proposed to study the effect of individual and household characteristics on the likelihood of falling behind one’s finances.

The analysis results show that traditional variables such as income, age, education, and health are statistically significant in explaining the variation in the likelihood of falling behind in one’s finances. In addition, the study shows that the volatility of income, saving habits, and an individual’s financial knowledge significantly contribute to explaining the probability of whether Americans fall behind in their finances or not. Based on the results, some recommendations would be to increase the exposure of Americans to financial knowledge, create incentives to increase their saving habits, and adopt measures to improve their health status. It might that the level of financial literacy be the reason of not making well-informed financial decisions that lead to financial well-being. Future research might investigate the causes and the intervention policies needed to overcome the lack of financial literacy among different groups of Americans.

## Data availability statement

Publicly available datasets were analyzed in this study. This data can be found at: https://www.consumerfinance.gov/data-research/research-reports/financial-well-being-america.

## Ethics statement

Ethical review and approval was not required for the study on human participants in accordance with the local legislation and institutional requirements. Written informed consent from the [patients/participants OR patients/participants legal guardian/next of kin] was not required to participate in this study in accordance with the national legislation and the institutional requirements.

## Author contributions

All authors listed have made a substantial, direct, and intellectual contribution to the work and approved it for publication.

## Conflict of interest

The authors declare that the research was conducted in the absence of any commercial or financial relationships that could be construed as a potential conflict of interest.

## Publisher’s note

All claims expressed in this article are solely those of the authors and do not necessarily represent those of their affiliated organizations, or those of the publisher, the editors and the reviewers. Any product that may be evaluated in this article, or claim that may be made by its manufacturer, is not guaranteed or endorsed by the publisher.

## Footnotes

^1^See￼ for example, Kavetsos et al. (2014) or Dolan et al. (2008) for a detailed survey of the literature about the topic.

^2^The misery index is an economic indicator obtained by adding the seasonally adjusted unemployment and inflation rates.
